# Supplementary material for: Comparative multiomics analysis of cell physiological state after culture in a basket bioreactor
Source: Sci Rep. 2022 Nov 23;12:20161. doi: 10.1038/s41598-022-24687-4 (PMC9686226; doi:10.1038/s41598-022-24687-4)

6037.1:154686448:154686764:+@NW_023666037.1:154686448:154686772:+@NW_023666037.1:154691950:15

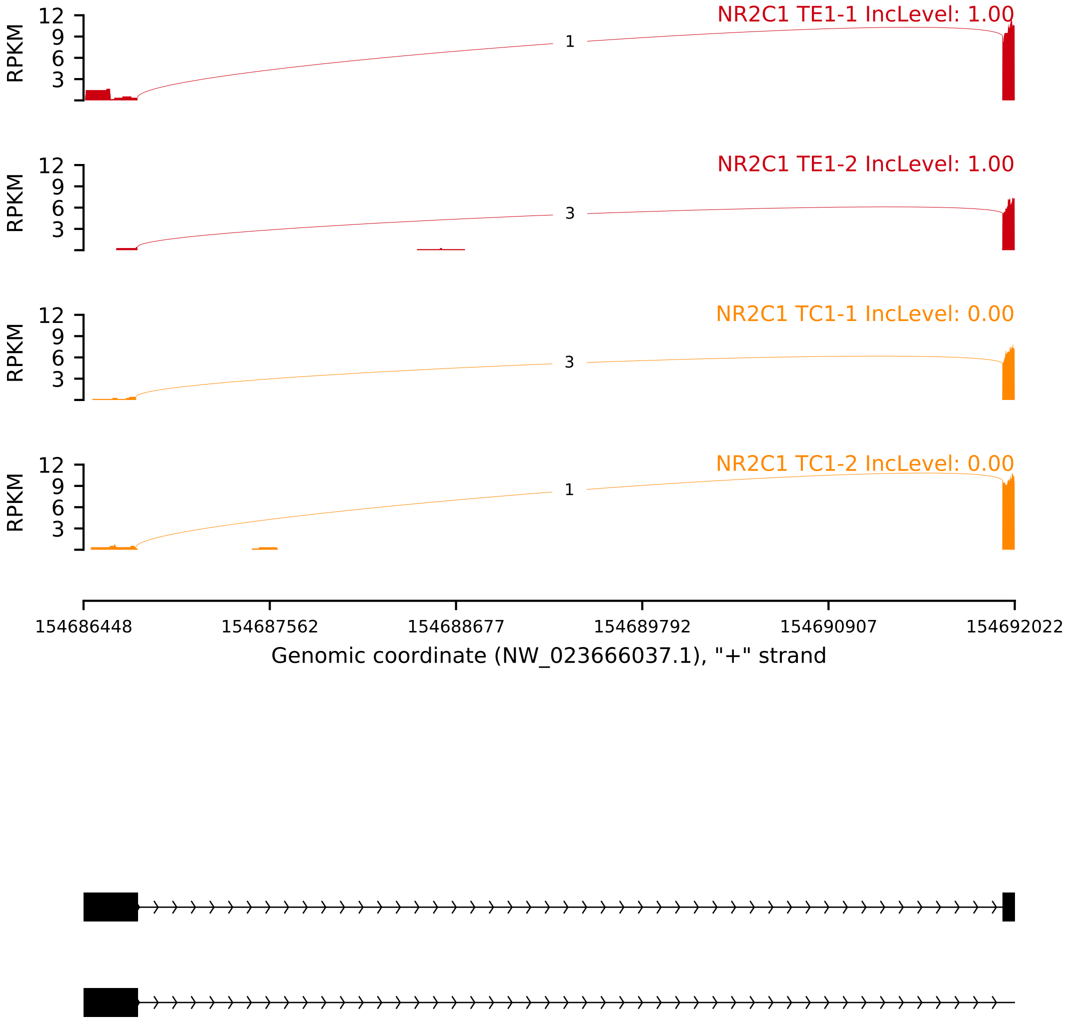

Supplement: Supplementary file 1 — Supplementary Information 1. [file 41598_2022_24687_MOESM1_ESM.zip › raw data/Transcriptomic raw data/7.AS/2.dasplot/TE1vsTC1/A5SS/10_NR2C1_NW_023666037.1_154686448_154686764_+@NW_023666037.1_154686448_154686772_+@NW_023666037.1_154691950_154692023_+.pdf]
